# Donor monocyte–derived macrophages promote human acute graft-versus-host disease

**DOI:** 10.1172/JCI133909

**Published:** 2020-07-27

**Authors:** Laura Jardine, Urszula Cytlak, Merry Gunawan, Gary Reynolds, Kile Green, Xiao-Nong Wang, Sarah Pagan, Maharani Paramitha, Christopher A. Lamb, Anna K. Long, Erin Hurst, Smeera Nair, Graham H. Jackson, Amy Publicover, Venetia Bigley, Muzlifah Haniffa, A.J. Simpson, Matthew Collin

**Affiliations:** 1 Human Dendritic Cell Laboratory, Institute of Cellular Medicine, Newcastle University, Newcastle upon Tyne, United Kingdom.; 2 Northern Centre for Bone Marrow Transplantation and; 3 NIHR Newcastle Biomedical Research Centre, Freeman Hospital, Newcastle upon Tyne Hospitals NHS Foundation Trust, Newcastle upon Tyne, United Kingdom.; 4 Institute of Cellular Medicine and; 5 Northern Institute of Cancer Research, Newcastle University, Newcastle upon Tyne, United Kingdom.

**Keywords:** Immunology, Transplantation, Bone marrow transplantation, Macrophages, Stem cell transplantation

## Abstract

Myelopoiesis is invariably present and contributes to pathology in animal models of graft-versus-host disease (GVHD). In humans, a rich inflammatory infiltrate bearing macrophage markers has also been described in histological studies. In order to determine the origin, functional properties, and role in pathogenesis of these cells, we isolated single-cell suspensions from acute cutaneous GVHD and subjected them to genotype, transcriptome, and in vitro functional analysis. A donor-derived population of CD11c^+^CD14^+^ cells was the dominant population of all leukocytes in GVHD. Surface phenotype and NanoString gene expression profiling indicated the closest steady-state counterpart of these cells to be monocyte-derived macrophages. In GVHD, however, there was upregulation of monocyte antigens SIRPα and S100A8/9 transcripts associated with leukocyte trafficking, pattern recognition, antigen presentation, and costimulation. Isolated GVHD macrophages stimulated greater proliferation and activation of allogeneic T cells and secreted higher levels of inflammatory cytokines than their steady-state counterparts. In HLA-matched mixed leukocyte reactions, we also observed differentiation of activated macrophages with a similar phenotype. These exhibited cytopathicity to a keratinocyte cell line and mediated pathological damage to skin explants independently of T cells. Together, these results define the origin, functional properties, and potential pathogenic roles of human GVHD macrophages.

## Introduction

Acute graft-versus-host disease (GVHD) affects up to 50% of patients receiving allogeneic bone marrow transplantation (BMT) and remains a leading cause of morbidity and mortality (1, 2). GVHD most commonly affects the skin, gut, and liver and may also contribute to idiopathic pneumonia syndrome (3). In animal models, donor T lymphocytes play an essential role in immune-mediated damage to host epithelium (4). In human GVHD, mononuclear infiltrates have been observed that include CD8^+^ T cells, CD4^+^ Th1, Th2, Th17 cells, and Tregs, although no pathognomonic effector subset has been observed in all patient cohorts (5–8). Despite the obvious importance of effector T cells, they may not be sufficient to mediate GVHD pathology (4). In almost all GVHD models, pathology occurs in the presence of neutrophils, monocytes, and other myeloid components that may infiltrate tissues and amplify local immune responses (9).

Animal models previously demonstrated that immunocompetent donor myeloid cells enhance GVHD, without specifying a particular cell type (10, 11). Macrophages have been implicated through observations that GVHD may be modulated by manipulation of the macrophage CSF (M-CSF) axis, although opposing effects have been reported, depending on the timing of interventions (12–15). Glucocorticoids also appear to reduce GVHD, at least partly through attenuation of macrophage responses (16), and in humanized mice, donor monocytes or DCs are absolutely required for xeno-GVHD (17). Knockout of the ATP receptor P2Y2 on recipient monocytes reduces GVHD lethality (18). Most recently, a specific role of T cell–derived GM-CSF was described in promoting the differentiation of effector macrophages (19).

In humans, a number of reports highlight an increase in myeloid cells bearing macrophage markers, showing that the level of infiltration correlates with clinical severity and outcome (7, 20, 21). However, as shown by high-resolution analysis, the myeloid cell compartment of human skin is highly complex, with discrete populations of classical DCs, monocyte-derived cells, and resident macrophages (22–26). These observations suggest that the nature of myeloid infiltrates cannot be adequately resolved using in situ microscopy; hence, their origin and immune functions in GVHD remain undefined.

The role of (recipient) myeloid cells in responding to danger signals is integral to most models of GVHD, but it is not known whether human GVHD infiltrates bearing macrophage markers are recipient or donor in origin and immunogenic or antiinflammatory in activity.

Although donor myelopoiesis usually dominates the peripheral blood compartment during GVHD, recipient dermal macrophages have very slow kinetics of turnover in humans (22) and potentially expand during inflammation (27). Macrophages are capable of mediating a wide spectrum of tolerogenic or pathogenic responses (28). By extrapolation from mouse models, macrophages are likely to promote GVHD. However, their ability to stimulate local effector T cells and mediate direct epithelial damage remains untested in humans.

Here, we employ direct methods of isolation and testing to show that acute GVHD lesions in human skin are dominated by CD11c^+^CD14^+^ myeloid cells with the genotype, phenotype, and transcriptional profiles of donor monocyte–derived macrophages. These cells have potent immunological functions that are likely to contribute to the pathogenesis of GVHD and may offer opportunities for therapeutic intervention.

## Results

**Figure 1:**
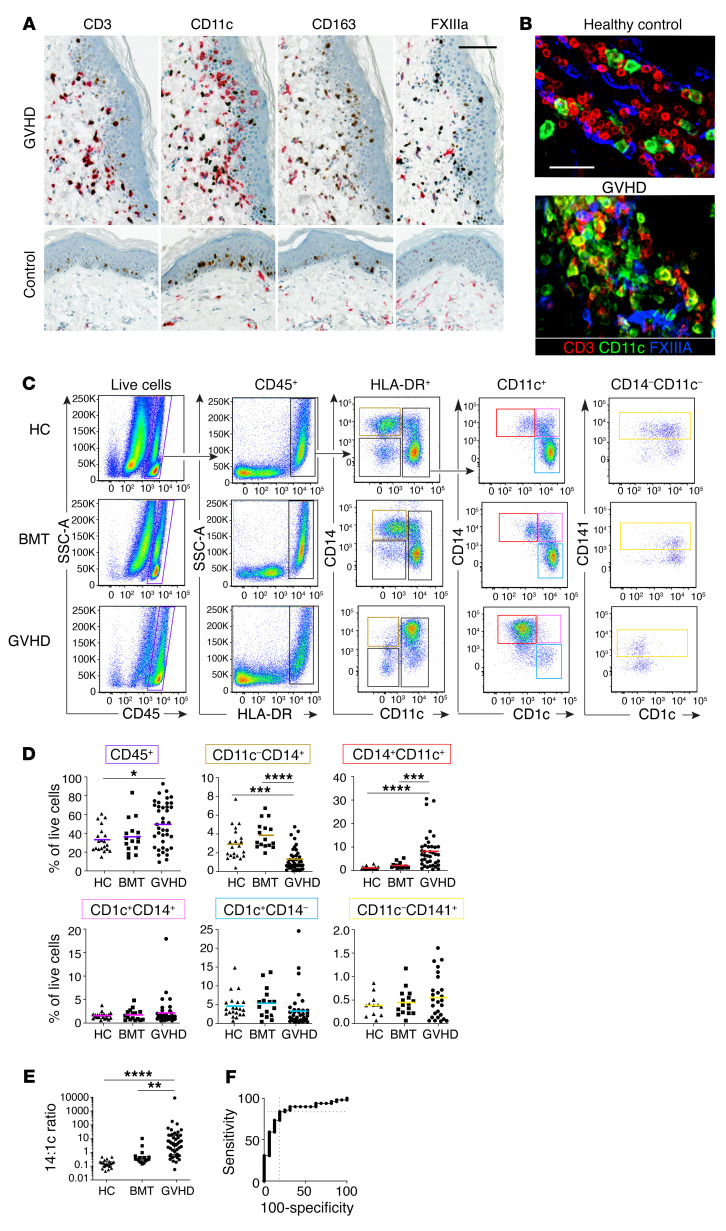
Mononuclear infiltrates in GVHD contain abundant CD14^+^CD11c^+^ myeloid cells. Microscopic and flow cytometric evaluation of cutaneous GVHD lesions. (**A**) Acute GVHD (top row) and healthy control skin (bottom row). Immunohistochemistry with antibodies to CD3, CD11c, CD163, and factor XIIIa (red chromagen) costained with antibody to Ki67 (brown chromagen). Scale bar: 100 μm. (**B**) Whole-mount immunofluorescence of dermis from healthy controls and patients with GVHD, as indicated with antibodies to CD3 (red), CD11c, (green), and FXIIIA (blue). Scale bar: 50 μm. (**C**) Enzymatically digested dermis analyzed by flow cytometry from patients with GVHD, patients without GVHD (BMT), or healthy controls (HC), as indicated. Starting from CD45^+^ mononuclear cells (purple gate), HLA-DR^+^ cells were gated as shown to arrive at CD11c^–^CD14^+^ resident macrophages (brown), CD11c^+^CD14^+^CD1c^–^ monocyte-macrophages (red), CD11c^+^CD14^+^CD1c^+^ double-positive cells (pink), CD1c^+^CD14^–^ cDC2 (cyan), and CD141^+^ cDC1 (yellow; from the CD14^–^CD11c^–^ gate). Representative samples of more than 60 experiments are shown. (**D**) Quantification of digested dermal mononuclear cells from patients with GVHD (*n* = 39), patients without GVHD (*n* = 16), or healthy controls (*n* = 21) as percentages of live cells. Mean + SEM for each group is shown. Groups were compared by 1-way ANOVA, and *P* values from Tukey’s multiple comparisons tests are shown. *P < 0.05; **P < 0.01; ***P < 0.001; ****P < 0.0001. (**E**) Ratio of CD11c^+^CD14^+^ cells to CD1c^+^CD14^–^ cells in digests of GVHD, BMT control, or healthy control dermis (14:1c ratio). Median and interquartile range for each group are shown. Groups were compared by Kruskal-Wallis test, and *P* values from Dunn’s multiple comparisons test are shown. (**F**) ROC curve analysis of 14:1c ratio in digested cells from GVHD versus BMT controls. AUC = 0.85. Maximal sensitivity and specificity occurred at a ratio of greater than 0.55.

In order to investigate the properties of myeloid cells in human cutaneous acute GVHD, mononuclear cells of the human dermis were defined by immunohistochemistry, immunofluorescence microscopy, and flow cytometry in healthy controls, BMT recipients without acute GVHD, and BMT recipients with GVHD (Supplemental Table 1 and Supplemental Table 2; supplemental material available online with this article; https://doi.org/10.1172/JCI133909DS1). BMT control patients without GVHD were biopsied on median day 83 after transplantation (range, 28–148 days). GVHD skin biopsies were taken at the onset of an acute onset erythematous rash, before initiation of therapy. Classical acute GVHD, immunosuppression withdrawal acute GVHD, and acute GVHD following donor lymphocyte infusion were all included. A pathological diagnosis of acute-type GVHD was confirmed by standard histological criteria in all cases, and patients with clinical or histological features of chronic GVHD were excluded. The median day of biopsy was day 53 (range, 13–304; Mann-Whitney U test, *P* = 0.27 compared with BMT controls). In situ analysis showed an increase in CD3^+^ T cells and CD11c^+^ myeloid cells in a perivascular and epidermal interface distribution in GVHD (Figure 1, A and B). The nature of the leukocytic infiltrate was also documented using 4-color immunofluorescence of whole-mount specimens. There was marked infiltration of perivascular spaces by CD11c^+^ cells that usually remained distinct from FXIIIA-expressing resident macrophages (ref. 22 and Figure 1B). Further comparison of CD11c, FXIIIA, and CD163 antigen expression by this approach is shown in Supplemental Figure 1, A–C.

The infiltrates of acute GVHD infiltrate were characterized by flow cytometry of single-cell suspensions. Gating on live singlets expressing CD45 and HLA-DR revealed side scatter (SSC) low lymphocytes and HLA-DR^+^ SSC high myeloid cells, as previously described (22, 25). Surprisingly, the proportion of cells falling in the lymphoid gate was not significantly increased in GVHD relative to BMT controls or healthy donors (Supplemental Figure 1, D and E). A relative expansion of IFN-γ–secreting CD4^+^ T cells was observed in GVHD skin relative to healthy controls, although this population was also elevated in BMT controls compared with healthy skin (Supplemental Figure 1F). Myeloid cells were further divided on the bivariate plot of CD14 versus CD11c, allowing identification of subsets previously described in healthy control skin without relying upon autofluorescence to capture resident macrophages (22–24, 26). Cells captured in the CD14^+^CD11c^+^ gate corresponded to cells captured in the autofluorescence negative CD14^+^ gate previously described in healthy control skin (25). The linkage between this gating strategy and previously identified myeloid cell populations is explained in Supplemental Figure 2, A and B.

In contrast to the modest changes in overall cellularity and T cell populations, CD11c^+^CD14^+^ myeloid cells were expanded more than 10-fold compared with healthy control skin or BMT skin without GVHD (Figure 1, C and D, and Supplemental Figure 1, A–C). This GVHD-related subset lacked CD1c expression and mapped to autofluorescence-negative CD14^+^ parameter space containing monocyte-macrophages in the steady state (25). Cells in the CD14^+^CD11c^–^ gate contained FXIIIA^+^CD163^+^ macrophages with high melanin content and autofluorescence, representing “fixed” or resident macrophages (22, 29, 30). These were relatively depleted in GVHD, as were classical DC2 (cDC2) (CD11c^+^ CD1c^+^CD14^–^) and cDC1 (CD141^+^ cells in the CD14^–^CD11c^–^ gate; Figure 1, C and D). The ratio of digested CD11c^+^CD14^+^ cells to CD1c^+^ cDC2 was markedly increased in GVHD (Figure 1E)

By ROC curve analysis, a ratio of more than 0.55 was 84% sensitive and 81% specific for the histological diagnosis of GVHD in skin biopsies after BMT (Figure 1F). Sequential biopsies showed resolution of the GVHD infiltrate in parallel with clinical improvement (Supplemental Figure 2, C and D).

**Figure 2:**
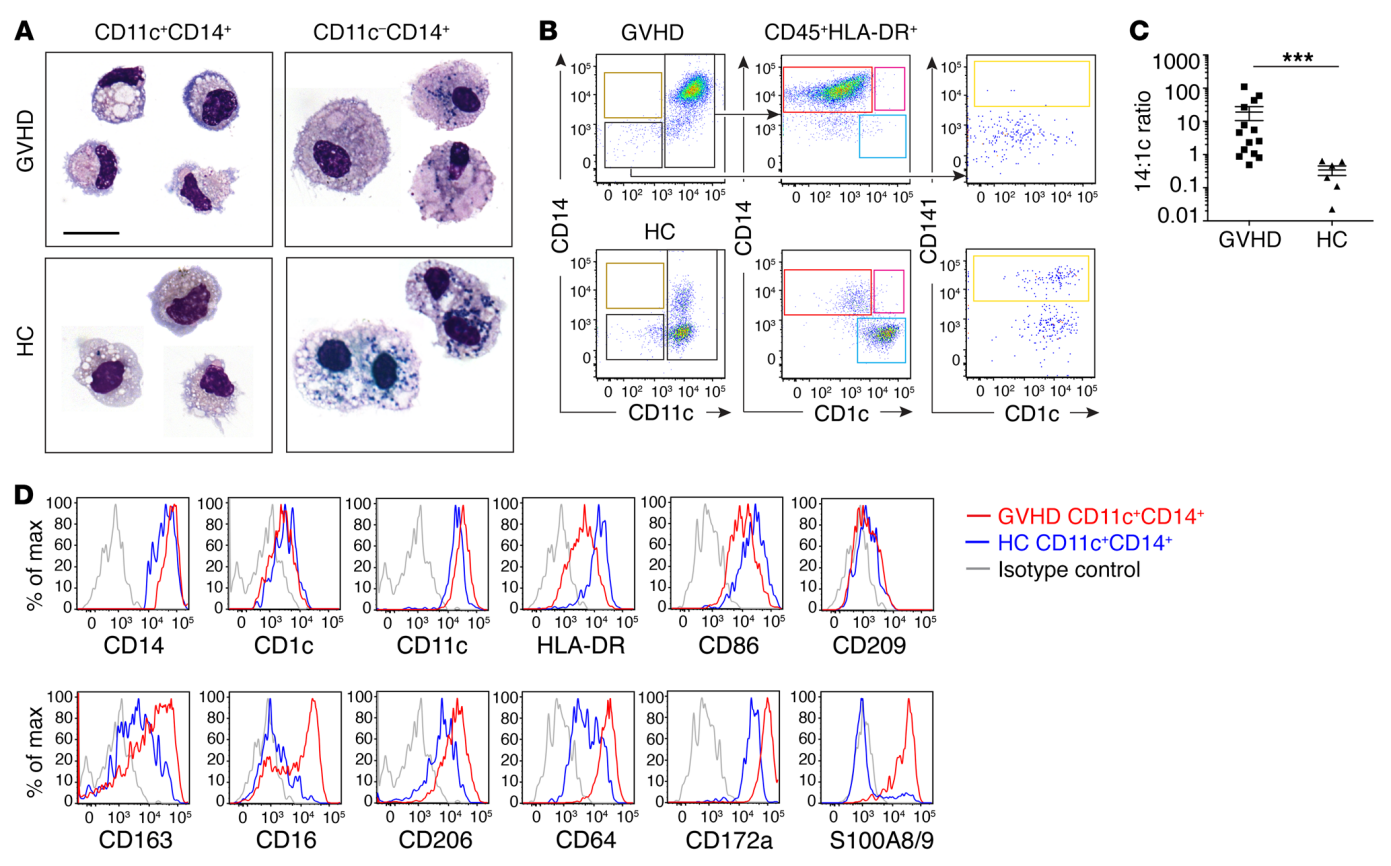
CD14^+^CD11c^+^ cells are small migratory macrophages with monocyte antigen expression. (**A**) May-Grünwald Giemsa–stained cytospins of CD11c^+^CD14^+^ and CD11c^–^CD14^+^ myeloid cells sorted from GVHD dermis and healthy controls. Representative cells from 2 to 4 concatenated images are shown. Scale bar: 20 μm. (**B**) Flow cytometry analysis of CD45^+^HLA-DR^+^ leukocytes migrating from explanted GVHD or healthy control skin over 48 hours in vitro. Gating as in Figure 1. (**C**) Comparison of CD14/CD1c ratio in migrating cells from GVHD skin (*n* = 14) and healthy controls (*n* = 6). Data are represented as mean ± SEM. ***P = 0.0002, Mann-Whitney *U* test. (**D**) Relative expression of selected antigens on CD11c^+^CD14^+^ cells migrating from GVHD skin (red line) or healthy control (blue line) compared with isotype control (gray line). Representative data from at least 3 donors are shown.

We sought to characterize the excess of CD11c^+^CD14^+^ cells observed in GVHD further, showing by morphology that they were small macrophages with eccentric dense nuclei, cytoplasmic vacuoles, and granules, distinct from larger, melanin-rich macrophages isolated from the CD14^+^CD11c^–^ gates (Figure 2A). They retained migratory capacity in vitro similar to that of their steady-state counterparts (ref. 25 and Figure 2B). An increase in the ratio of migratory CD11c^+^CD14^+^ cells to CD1c^+^ cDC2 was also observed in GVHD (Figure 2C), as seen in digested preparations (Figure 1E). CD11c^+^CD14^+^ GVHD cells expressed common macrophage antigens (CD163, CD64, CD206, and CD209), but showed upregulation of monocyte-associated antigens (CD172a, S100A8/9, CD16 (Figure 2D).

**Figure 3:**
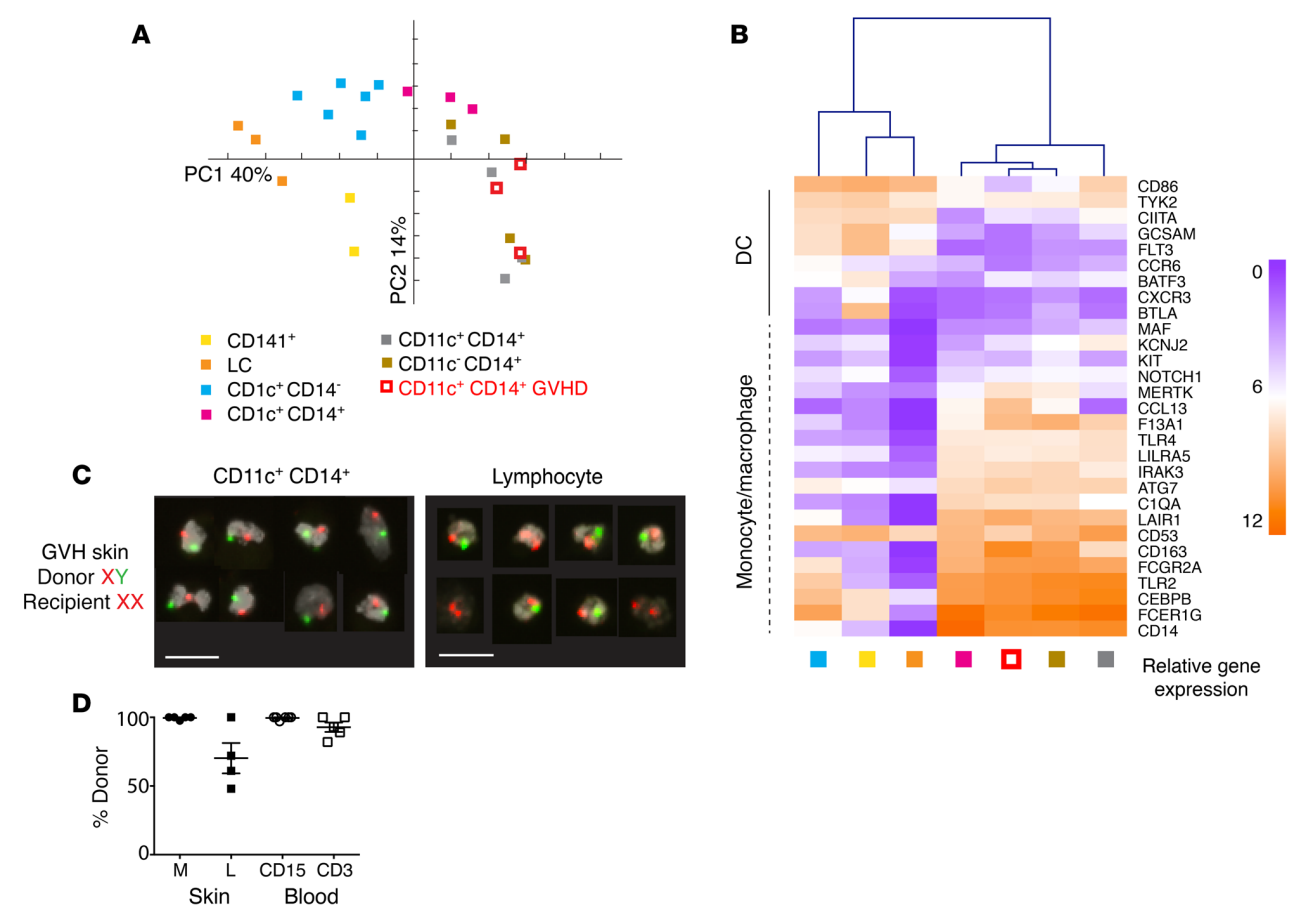
CD14^+^CD11c^+^ myeloid cells are donor-derived macrophages. (**A**) PCA of immune gene expression by CD11c^+^CD14^+^ GVHD cells and 6 myeloid subsets from healthy control skin. Myeloid cells were sorted from healthy control skin as described in Figure 1 and are annotated accordingly. (**B**) Heatmap showing unsupervised clustering of CD11c^+^CD14^+^ cells from GVHD skin and myeloid cells derived from healthy control skin. Mean log_2_ expression for each subset is shown. *n* = 2 for CD141^+^; *n* = 3–6 for all other subsets. (**C**) Example of FISH showing the XY genotype of GVHD macrophages (CD11c^+^CD14^+^) and lymphocytes sorted from a female recipient transplanted with a male donor. A single field viewed at ×10 magnification was concatenated to show 8 representative cells per image. Scale bars: 20 μm. (**D**) Percentages of donor origin analyzed by XY FISH of macrophages (M) and lymphocytes (L) sorted from lesional GVHD skin compared with CD15^+^ myeloid cells (CD15) and lymphocytes (CD3) sorted from paired blood samples.

**Figure 4:**
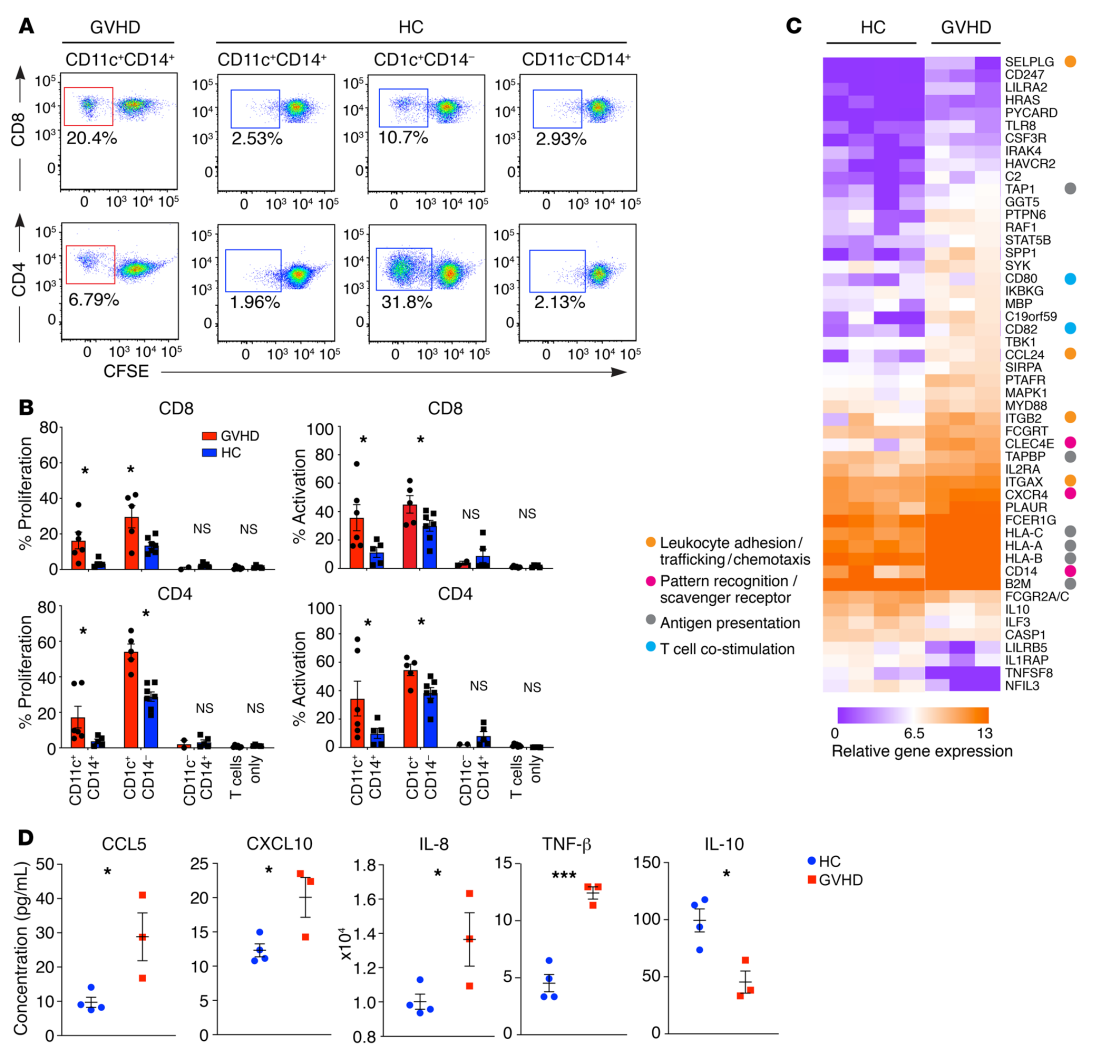
GVHD macrophages activate allogeneic T cells. (**A**) Proliferation of allogeneic CD4^+^ and CD8^+^ cells estimated by CFSE dilution after coculture with DC and macrophage subsets sorted from GVHD or healthy controls. (**B**) Summary of T cell proliferation (percentage of CFSE dilution) and activation (percentage of CD69^+^CD8^+^ T cells and percentage of HLA-DR^+^ CD4^+^ T cells) from *n* = 3 experiments. *P < 0.05, unpaired *t* test. (**C**) Heatmap of genes differentially expressed between CD11c^+^CD14^+^ monocyte-derived macrophages sorted from healthy control skin (*n* = 4) and GVHD skin (*n* = 3) with fold difference in log_2_ gene expression of greater than 1.3. *P* < 0.05, unpaired *t* test. Annotations show functional attributes of genes (based on Entrez Gene summaries) upregulated in GVHD macrophages. (**D**) CD11c^+^CD14^+^ monocyte–derived macrophages sorted from GVHD (*n* = 3) and healthy control dermis (*n* = 4) were stimulated with LPS in culture over 10 hours. Chemokine and cytokine production were quantified in supernatants by Luminex assay. Data are represented as mean ± SEM. *P < 0.05; ***P < 0.001, unpaired *t* test.

In order to define the ontogeny of CD11c^+^CD14^+^ cells relative to known populations of macrophages and DCs, GVHD and steady-state populations were sorted and expression of 609 immunology-related genes was surveyed by NanoString. By principal component analysis (PCA), CD11c^+^CD14^+^ GVHD cells were segregated with steady-state monocyte-macrophages and resident dermal macrophages, away from DC populations (Figure 3A). Focusing on a previously defined subset of 29 genes that distinguish between DCs and monocytes or macrophage lineages (25), CD11c^+^CD14^+^ GVHD cells were clustered with steady-state monocyte-macrophages and resident macrophages in an unsupervised analysis (Figure 3B). Genotype analysis by XY FISH in sex-mismatched transplants showed a median of 98%–100% donor origin of CD11^+^CD14^+^ GVHD macrophages, equal to the level of blood myeloid chimerism (Figure 3, C and D). Based on these results, we conclude that CD11c^+^CD14^+^ myeloid cells in GVHD are donor monocyte–derived macrophages. Recipient T cells were present in the dermis in 3 out of 4 patients at the time of GVHD. Although myeloid cells have previously been described in human GVHD by histology, their functional properties have not been directly tested. Steady-state CD14^+^ monocyte–derived macrophages are not potent allostimulators compared with dermal CD141^+^ cDC1 and CD1c^+^ cDC2 (22, 23). In contrast, GVHD macrophages were capable of stimulating T cell proliferation and expression of activation antigens to the levels associated with steady-state DC populations (Figure 4, A and B). Gene expression profiling of 2000 to 5000 sorted cells revealed upregulation of allostimulatory functions that included antigen presentation (*HLA*, *TAP1*), recruitment (*CCL24*) and stimulation of lymphocytes (*CD82*), stimulation of proinflammatory cytokines (*SPP1*), and leukocyte extravasation (*SELPLG*) (Figure 4C). Differential expression of several key chemokines and cytokines was also revealed at the protein level, including CCL5/RANTES, CXCL10, IL-8, TNF-β, and IL-10 (Figure 4D).

**Figure 5:**
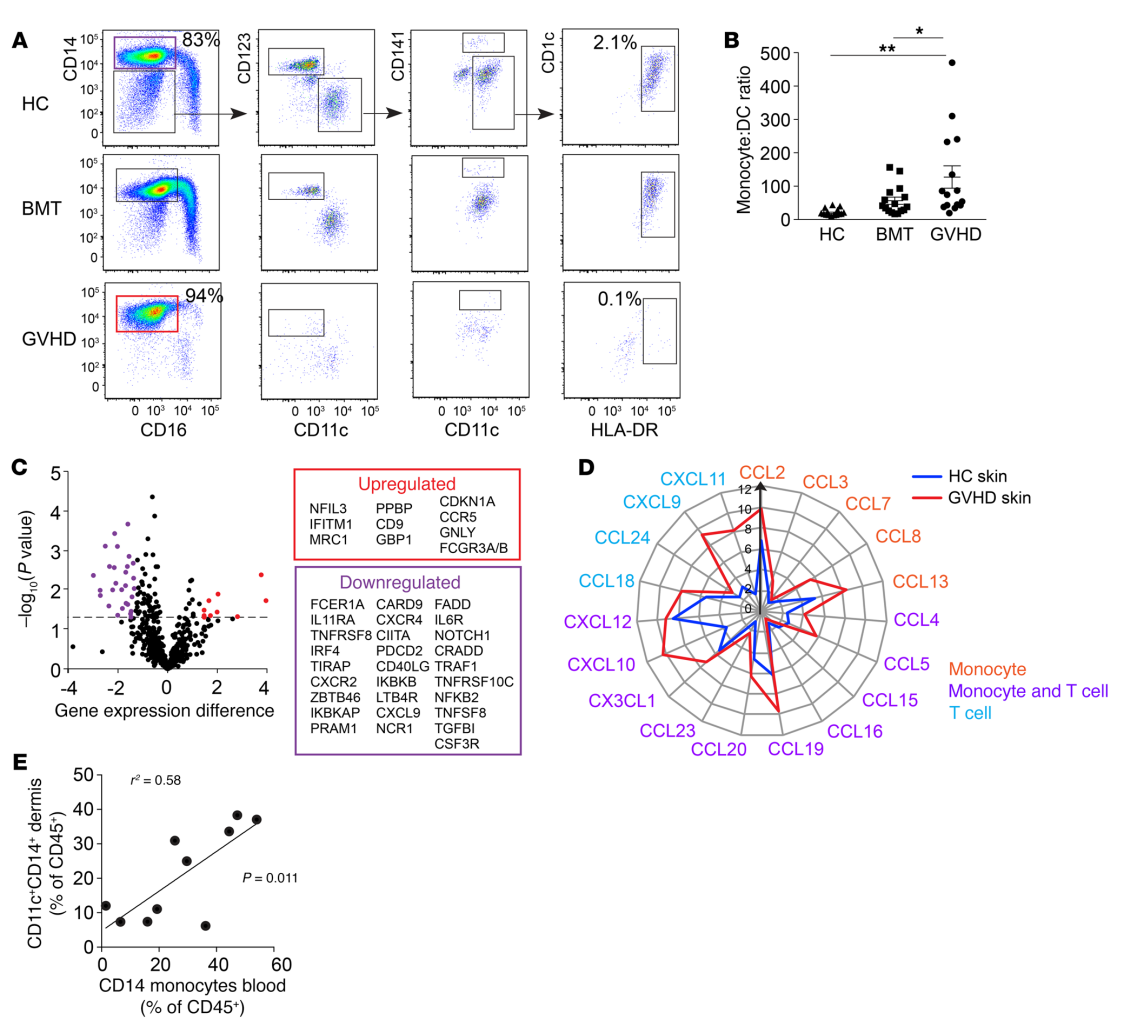
Monocytes are poised to differentiate into GVHD macrophages. (**A**) Comparison of PBMCs of healthy control, transplant patients without GVHD, and patients with GVHD. CD3^–^CD4^+^HLA-DR^+^ monocyte and DC populations were divided into CD14^+^ classical monocytes and CD14^–^CD16^–^ DCs, including CD123^+^CD11c^lo^ pDC, CD141^+^ cDC1, and CD1c^+^ cDC2. Representative examples of 10 experiments are shown. Frequencies of gated CD14^+^ monocytes and CD1c^+^ cDC2 are indicated as percentages of HLA-DR^+^ cells. (**B**) Ratio of CD14^+^ monocytes to CD1c^+^ cDC2 in blood of GVHD patients (*n* = 15), BMT controls (*n* = 16), and healthy controls (*n* = 15) analyzed by flow cytometry, as shown in **A**. Data are represented as mean + SEM. *P < 0.05; **P < 0.01, 1-way ANOVA and Tukey’s multiple comparison tests. (**C**) Genes differentially expressed between healthy control monocytes and GVHD classical monocytes (upregulated in red and downregulated in purple) at fold difference in log_2_ gene expression of greater than 1.3 and *P* < 0.05. Cells sorted from *n* = 6 GVHD and *n* = 3 HC individuals. (**D**) Radial plot showing mean expression of chemokine genes in whole skin from patients with GVHD (red line; *n* = 10) and healthy controls (blue line; *n* = 6). Expression of the corresponding receptors by monocyte, T cell, or both is indicated. (**E**) Correlation between blood CD14^+^ monocyte frequency and CD11c^+^CD14^+^ content of GVHD dermis in paired blood and skin samples from 10 patients with GVHD. Statistical test by linear regression.

The presence of prominent monocyte-derived populations in human GVHD prompted us to examine the peripheral blood for evidence of altered myelopoiesis or priming of monocytes. Classical monocytes were enriched in patients with GVHD, especially in proportion to CD1c^+^ cDC2, as described in GVHD skin (Figure 5, A and B). Analysis of differential gene expression between the monocytes of patients with GVHD and healthy controls showed upregulation of monocyte chemoattractant receptor *CCR5* and *MRC1* (macrophage mannose receptor, CD206), *FCGR3A/B* (Fc receptor/CD16), *GNLY* (granulysin), and IFN-response genes *IFITM1* and *GBP1* (Figure 5C). Downregulation of a large module of genes was associated with DC differentiation, such as *FCER1A*, *IRF4*, *ZBTB46* and *CIITA*.

In addition to monocyte priming, whole-skin gene expression of GVHD-affected skin showed prominent upregulation of monocyte (and T cell) chemokine receptor-ligand pairs (Figure 5D). The proportion of CD14^+^CD11c^+^ GVHD macrophages found in affected skin mirrored the relative expansion of CD14^+^ monocytes in the blood (Figure 5E).

**Figure 6:**
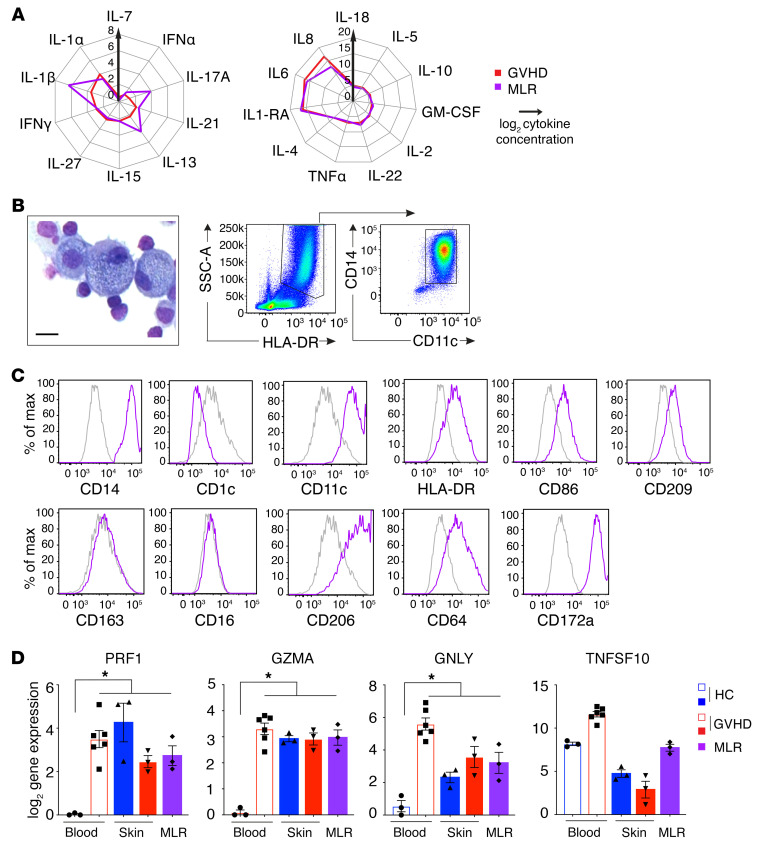
Allostimulated monocytes resemble GVHD macrophages. (**A**) Radial plots of cytokine quantity in supernatants from GVHD explants cultured for 48 hours (red line) and BMT donor-recipient MLRs cultured for 7 days (purple line). Lines show mean cytokine concentration from *n* = 12 (GVHD) and *n* = 6 (MLR) experiments. IL-9, IL12p70, IL-23, IL-31, and TNF-β are not shown because they were not detected in any specimens. (**B**) May-Grünwald Giemsa cytospin morphology, scatter properties, and CD11c/CD14 expression by MLR macrophages, isolated on day 7. Scale bar: 20 μM. (**C**) Expression of selected antigens, previously used to define GVHD macrophages, by allostimulated CD11c^+^CD14^+^ cells from BMT donor-recipient MLRs (specific staining in purple; isotype control in gray). Representative histograms from more than 3 analyses are shown. (**D**) Expression of cytotoxic effector genes in CD14^+^ blood monocytes, skin CD11c^+^CD14^+^ cells, and MLR macrophages. Columns indicate mean and bars SEM of *n* = 3–6 values; *P < 0.05, Kruskal-Wallis test with *P* values from Dunn’s multiple comparison tests is shown.

The preceding data suggest that GVHD macrophages are donor derived from blood monocytes and achieve a higher state of functional activation than their steady-state counterparts. Further evidence of their likely function in GVHD was sought by deriving allostimulated macrophages from monocytes and testing their functional properties. HLA-matched donor and recipient blood was taken before transplantation, and PBMCs were stored in order to prepare mixed leukocyte reactions (MLRs). The cytokine milieu of an HLA-matched MLR was similar to that observed when GVHD skin was cultured (Figure 6A), and a prominent population of macrophages appeared with a phenotype similar to that of GVHD macrophages (Figure 6, B and C). Between monocytes and MLR-derived macrophages, 118 transcripts were differentially expressed (FDR ≤0.05; Supplemental Table 3 and Supplemental Figure 3). *MRC1*, *CCR5*, and *PPBP*, upregulated in GVHD macrophages in vivo, were also highly upregulated in MLR macrophages. MLR-activated macrophages also expressed cytotoxic molecules perforin, granzyme A, granulolysin, and TRAIL, similarly to GVHD macrophages (Figure 6D). Many of these products were already upregulated in CD14^+^ monocytes isolated from the blood of patients with GVHD compared with healthy control monocytes (Figure 6D).

**Figure 7:**
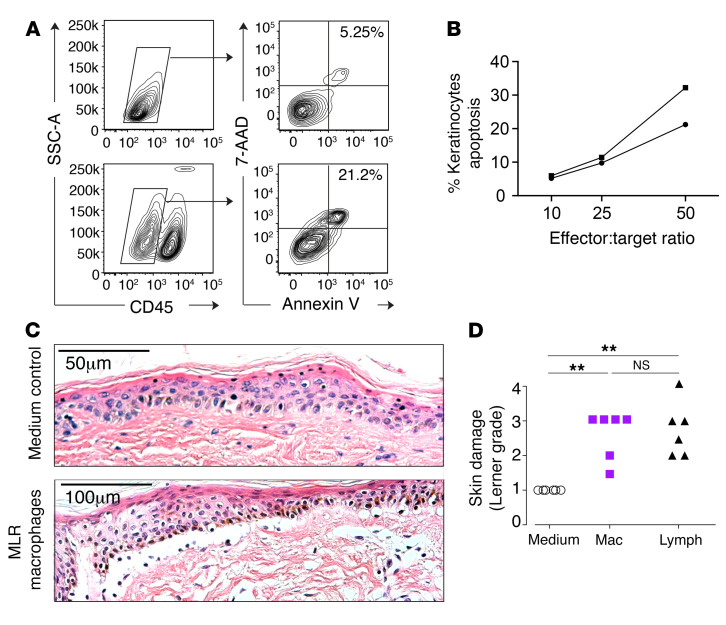
Cytoxicity of alloactivated macrophages in vitro. (**A**) Direct cytotoxicity of MLR macrophages to the keratinocyte cell line HaCaT was assessed by coculture of HaCaT and MLR macrophages at a range of effector/target ratios for 5 hours. Keratinocytes were identified as CD45^–^ cells by flow cytometry, and the proportion of dead keratinocytes was quantified by annexin V and 7-AAD staining. Representative flow cytometry plots from keratinocytes alone (top row) and keratinocytes cultured with MLR macrophages at a 50:1 ratio (bottom row). (**B**) Quantitation of keratinocyte apoptosis versus effector/target ratio in 2 independent experiments. (**C**) Experiments using the skin-explant model of GVHD (see Methods for details). MLR outputs were sorted to yield macrophages and lymphocytes and cocultured with shave biopsies of recipient skin for 3 days. Explants were fixed and stained with H&E. Representative images from explants cultured for 3 days in control medium or medium with MLR macrophages, as indicated. (**D**) Summary of histological damage to the dermoepidermal junction graded on the Lerner scale from 6 independent experiments. **P < 0.01, Kruskal-Wallis and Dunn’s multiple comparison tests. Mac, macrophages; lymph, lymphocytes.

The expression of cytotoxic molecules prompted us to test the possibility that MLR-activated macrophages might mediate cytotoxicity to epidermal cells. We observed that MLR-activated macrophages were directly cytotoxic to a keratinocyte cell line in vitro in a dose-dependent manner (Figure 7, A and B). In order to test a setting more relevant to GVHD, we adapted the in vitro skin-explant model. When a small explant of intact skin is exposed to a clone of minor-histocompatibility antigen-specific T cells, GVHD-like epidermal pathology is observed in an HLA-restricted and antigen-specific manner (31). GVHD pathology is also observed, in proportion to HLA matching and sex differences, when recipient skin is exposed to donor leukocytes presensitized to recipient antigens in an MLR (32). Although it has been assumed that GVHD pathology in vitro is exclusively mediated by T cells in the MLR, we were surprised to observe nearly equivalent cytopathic effects when the “donor” MLR was sorted into macrophage and T cell components (Figure 7, C and D).

## Discussion

In this study, we have defined the role of myeloid cells in human cutaneous acute GVHD by characterizing mononuclear infiltrates from primary tissue, isolating the dominant myeloid cell, and defining its origin, transcriptional profile, and functional properties. The data indicate that human GVHD lesions are highly infiltrated with donor monocyte–derived macrophages with enhanced allostimulatory activity and the potential to mediate epidermal pathology.

Myeloid cells found in GVHD have previously been characterized as macrophages based on histopathology describing a small number of surface antigens. These studies lack further details of the biological characteristics or potential pathogenic role of macrophages in GVHD (7, 20–22). Indeed, evidence that macrophages enhance local effector immune functions is surprisingly hard to find in any scenario of inflammation in human tissues. Where they have been isolated from primary human tissues, their function has been described as regulatory, in comparison with that of DCs (33, 34). Our findings that GVHD macrophages have functional attributes capable of promoting GVHD provide an important corroboration of recent mouse models describing the dependence of GVHD pathology upon donor myeloid cells activated by T cell–derived GM-CSF (19).

Numerically, macrophages show the greatest fold increase in GVHD of any mononuclear cell and constitute the most consistent “cellular signature” of acute GVHD relative to those of recipients without GVHD or healthy control skin. The macrophage/DC ratio is sensitive and specific relative to BMT control skin without GVHD, increasing more than 100-fold in the presence of GVHD. Previous studies of human GVHD have placed emphasis upon the potential existence of a pathognomonic subset of T cells in GVHD, although none has been consistently identified (6–8). Unlike animal models of BMT in which additional splenic T cells are added to initiate GVHD (4), human BMT recipients are typically severely lymphopenic owing to the routine use of T cell–depletion strategies, calcineurin inhibitors, and antimetabolites, such as methotrexate. Numerically, the T cell infiltrate is surprisingly modest and insignificantly different from that of healthy human skin, especially in classical early acute GVHD. The observations that MHC class I and II mismatches both increase the risk of GVHD and that CD4 or CD8 selective depletion does not abrogate GVHD are in keeping with multiple pathways of T cell alloreactivity that may vary from patient to patient (4). The striking feature is that all appear to result in the profound recruitment of inflammatory macrophages.

Here, GVHD macrophages were defined as CD11c^+^CD14^+^ cells based on the most direct means of distinguishing the infiltrate from autofluorescent CD11c^–^ resident macrophages by flow cytometry. Several lines of evidence point to a monocyte origin; most notably, they are donor derived and therefore unlikely to arise by proliferation of resident-recipient macrophages. Additional staining demonstrated high expression of monocyte antigens S100A8/A9 and SIRPA (CD172), consistent with recent emigration of monocytes from the blood (35, 36). NanoString gene expression analysis confirmed transcription of a core set of macrophage-related genes, including *MAF*, *MERTK*, *F13A1*, *CD163*, and *CD14*. Although GVHD macrophages have higher expression of monocyte antigens and a number of important functional differences, they are most closely related to CD11c^+^CD14^+^ dermal cells found in steady-state tissues and previously reported to have a transient, monocyte origin (25). Enhanced monocyte priming and recruitment to tissues is also suggested by the phenotypic activation of peripheral blood monocytes, previously reported in patients with active GVHD (37–39). We observed a similar phenomenon in the expression of cytotoxic genes by GVHD monocytes compared with those from healthy controls. In corroboration, we also observed that monocytes differentiating into macrophages in HLA-matched MLRs had phenotypes and functional properties similar to those of GVHD macrophages.

The data indicate that single surface markers previously used to define GVHD macrophages by histology often have variable expression under more detailed scrutiny. In keeping with previous reports (7, 20–22), CD163 was detectable by flow cytometry, but was less consistent than CD11c^+^ in identifying GVHD infiltrates by immunohistochemistry. CD163 is expressed by resident macrophages (40) and chronic inflammatory macrophages found in psoriasis (41), celiac disease (42, 43), and Crohn’s disease (44). In celiac disease, acute gluten challenge induces an increase of CD11c^+^CD14^+^ cells with modest expression of CD163 (43), reminiscent of the population we describe in GVHD, suggesting that similar pathways of inflammatory myeloid cell recruitment operate in other conditions. Higher CD163 expression was associated with longer intervals after transplant, consistent with previous descriptions of abundant CD163 expression in advanced GVHD lesions (20, 21). These findings are also in keeping with the original characterization of CD163 (clone RM3/1) as a “late-phase” macrophage antigen with more delayed kinetics of expression (45).

GVHD macrophages demonstrated enhanced T cell–stimulatory functions compared with steady-state CD11c^+^CD14^+^ cells, including greater expression of pattern recognition, leukocyte adhesion and trafficking, antigen presentation and T cell–costimulation genes, production of chemokines, and capacity to stimulate allogeneic T cell proliferation. Ideally, these functions would have been compared with those of CD11c^+^CD14^+^ cells isolated from BMT controls, but this was not possible owing to the paucity of these in small clinical biopsies of skin unaffected by GVHD, as shown in Figure 1. However, even steady-state CD11c^+^CD14^+^ cells already demonstrate upregulation of cytotoxic molecules compared with blood monocytes, suggesting that they exist in a poised state potentially governed by mediators that are further upregulated during GVHD, such as IFN-γ (46). The potential of macrophages to mediate direct cytotoxic effects is described in classical studies, but until recently, it has received little attention in the field of GVHD. Early studies showed that GVHD induced priming of macrophages, resulting in direct cytotoxicity following LPS challenge (47), and subsequent work elaborated on the secretory properties of activated macrophages (48). However, contemporary models of GVHD present the contribution of macrophages to GVHD almost exclusively in terms of sensing danger and enhancing accessory cell function (9–11). It was not possible to harvest sufficient GVHD macrophages directly from biopsies to test their effector function, so we generated allostimulated macrophages from monocytes in HLA-matched MLRs as a surrogate. MLR-stimulated macrophages were capable of mediating direct cytopathicity with a cell line and, surprisingly, caused a degree of immunopathology similar to that of T cells in the skin-explant model of GVHD. Although the latter lacks all the complexity of GVHD in vivo, it is the only fully human system amenable to manipulation. Furthermore, the degree of pathological damage consistently reflects levels of major and minor histocompatibility antigen matching and has been used previously to dissect HLA-restricted antigen-specific GVHD responses (31, 32). This result revises the assumption that T cells are the only relevant effectors when the MLR product is added to explanted skin.

In classical animal models of GVHD, myelopoiesis is invariably present during the effector phase of GVHD, even when the sole instigator of GVHD is a T cell clone directly targeted to epithelium (49, 50). Investigators have now revealed nonredundant functions for myeloid cells in GVHD pathogenesis (12–15, 17, 19). The conceptual advance that T cells are necessary, but may not be sufficient, for GVHD has important therapeutic implications. Unlike T cells in the human adult, which may take more than 2 years to recover after transplantation, myeloid cells are continuously generated, and rapid immune reconstitution is possible following myeloid-targeted interventions. The ability to isolate discrete mechanisms that govern the infiltration of tissues by myeloid cells, such as GM-CSF dependence, may also offer a means of minimizing GVHD without compromising graft-versus-leukemia (GVL) (19).

As described in the accompanying manuscript by Divito et al. (51), host tissues affected by GVHD, such as the skin and gut, contain a notable proportion of host-resident T cells that survive for many months and are found in an activated state in association with donor macrophages. Our results support the conclusion that donor-derived macrophages have enhanced antigen-presenting functions that could enable the activation of residual host T cells, resulting in host-versus-graft responses that may be indistinguishable from GVHD clinically. Recent observations in patients with host-versus-graft mismatches are consistent with this. A proportion of HLA-DP mismatching occurs exclusively in the host-versus-graft direction (heterozygous donor to homozygous recipient), and surprisingly, these patients have a high incidence of grade I GVHD (our unpublished observations). Further studies will be required to determine whether the marked difference in outcome between low-risk grade I acute GVHD and “clinically significant” grades II–IV acute GVHD reflects fundamental differences in mechanism. It is entirely plausible that many patients experience a self-limiting reaction of host T cells, resulting in skin-limited disease responsive to topical corticosteroids, while the canonical recruitment and activation of donor T cells only comes into play in higher grade disease. We have shown that donor macrophages are capable of performing both antigen-presenting and cytotoxic functions, but these may also be differentially involved depending on whether inflammation is primarily driven by recipient or donor T cells. Divito et al. observe that skin and gut both have prominent populations of resident T cells, and it will be important to explore the potential of donor macrophages to activate host T cells in gut GVHD (51).

The evidence that donor-derived macrophages perform essential functions in GVHD in both mice and humans contrasts with continuing uncertainty over the role played by recipient antigen-presenting cells. Although it is possible to construct mouse models in which GVHD depends solely on recipient myeloid cells or specific populations such as Langerhans cells (52–54), other models show that Langerhans cells (55), or indeed any hematopoietic antigen-presenting cell, are not required for GVHD (56). In human correlative studies, GVHD promotes donor myeloid cell engraftment, so the occurrence of GVHD is invariably associated with a loss of recipient antigen-presenting cells (57). Although human Langerhans cells are self-renewing (29, 58, 59), this potential is insufficient for maintaining them after transplantation; even with nonmyeloablative conditioning, they become almost fully donor derived in about 3 months (60).

In summary, the results presented here demonstrate that GVHD lesions contain abundant donor macrophages, likely to be derived from activated circulating classical monocytes. GVHD macrophages secrete chemokines, stimulate T cells, and mediate direct cytotoxicity. Together, these results shed light on human macrophage functions that are exploitable for the prevention and treatment of GVHD.

## Methods

### Human subjects.

Sequential patients undergoing allogeneic BMT were recruited from the Northern Centre for Bone Marrow Transplantation over a 3-year period between 2013 and 2016. Skin shaves of 5 to 15 mm^2^ were obtained from BMT recipients using 1% lidocaine local anesthesia and a DermaBlade (AccuTec Blades). Biopsies were performed at the onset of patient rashes clinically compatible with GVHD. An independent clinical pathologist provided diagnosis and histological grading of GVHD. BMT recipient controls with no evidence of any rash were biopsied when seen for routine assessments at day 28 or day 100 after transplant. Biopsies were transported in serum-free medium (X-VIVO, Lonza) and analyzed within 24 hours. An independent clinical pathologist provided diagnosis and histological grading of GVHD in controls and GVHD biopsies. Healthy control skin was obtained from patients undergoing mammoplasty or abdominoplasty, as previously described (22). Specimens comparable to clinical biopsies were obtained by immobilizing skin strips on a cork block covered with sterile silicon and performing skin shave biopsies.

### Cell lines and mononuclear cells.

Unless stated otherwise, all cells were cultured in RPMI 1640 (Lonza) with 100 IU/mL penicillin, 10 μg/mL streptomycin, 2 mM l-glutamine (Invitrogen), and 10% heat-inactivated FBS (Sera Lab). MLR macrophages were generated from coculture of PBMCs from HLA-matched BMT donor and recipient pairs. Recipient PBMCs were irradiated (20 Gy) and used as stimulators for donor PBMCs at a 1:1 ratio. Cultures containing 1 to 5 × 10^7^ donor PBMCs were maintained in RPMI 1640 with 10% human AB serum (MilliporeSigma) for 7 days. HaCaT cells were obtained from Accegen.

### Enzymatic digestion of skin biopsies.

Skin shave biopsies were used whole or split into dermis and epidermis by treatment with dispase 0.5–1.0 mg/mL in RPMI for 60 to 90 minutes at 37°C (Gibco, Thermo Fisher Scientific) before digestion in medium with 1.6 mg/mL type IV collagenase (Worthington) for 12 to 16 hours at 37°C in RPMI with 10% heat-inactivated FCS. Gentle dissociation and passage through a 100 μm filter generated single-cell suspensions.

### Migration of cells from skin biopsies.

For some experiments, an enzyme-free preparation of leukocytes was required. Skin was cultured as above, but without collagenase. After 48 hours, migratory leukocytes were harvested from the supernatant. Supernatants were stored at –80°C and later used for cytokine analysis by Luminex assay (see below)

### Immunohistochemistry.

Formalin-fixed and paraffin-embedded skin shave biopsies from GVHD diagnostic material were used. Sections of 4 μm were made. Antigen retrieval and staining were performed using the BenchMark autostainer (Ventana). CD3, CD163, CD11c, factor XIII, and Ki67 primary antibodies and the ultraView Detection Kit were used (Roche).

### Fluorescence microscopy.

Antibodies used for microscopy are listed in Supplemental Table 4. Sheets of 200 μm of whole skin were fixed in 2% paraformaldehyde (MilliporeSigma) and 30% sucrose (MilliporeSigma) in PBS (Gibco, Thermo Fisher Scientific) for 12 to 18 hours at 4°C, washed in PBS, and stored at 4°C until staining. Specimens were blocked in 0.5% BSA (MilliporeSigma) with 0.3% Triton X-100 (Thermo Fisher Scientific) in PBS and stained at 4°C for 12 to 18 hours with the following primary/secondary combinations: CD11c-biotin/streptavidin Cy5; factor XIII/donkey anti-sheep Alexa Fluor 647; CD3/donkey anti-mouse Alexa Fluor 488. Sections were immersed in DAPI-containing mounting medium for 12 to 18 hours at 4°C, then visualized on a Zeiss Axioimager Z2 using the Apotome function and Axiovision, version 4.8, software.

### Flow cytometry analysis and sorting.

Antibodies used for flow cytometry are listed in Supplemental Table 4. DAPI was added for dead cell discrimination (MilliporeSigma). Flow cytometry analysis and sorting were performed using BD FACS Canto II, BD Fortessa X20, BD FACS Aria II, and BD FACS Fusion running FACSDiva, version 7, and analyzed with FlowJo, version 10 (Tree Star).

### X/Y FISH.

Sorted cells were spun onto slides, fixed in methanol and acetic acid, and prepared with dual-labeled XY FISH probes on a ThermoBrite System (Abbott) in accordance with the manufacturer’s instructions.

### Gene expression by NanoString.

Gene expression was quantified by NanoString, using the Human Immunology, version 2, panel with a custom 30-gene add-on targeting monocytes, macrophages, and DC genes (*ASIP*, *C19orf59*, *CCL17*, *CD1C*, *CD207*, *CLEC10A*, *CLEC9A*, *CLNK*, *COBLL1*, *CXCL5*, *DBN1*, *F13A1*, *FGD6*, *FLT3*, *GCSAM*, *GGT5*, *MKI67*, *LPAR2*, *LYVE1*, *MAFF*, *MERTK*, *NDRG2*, *PACSIN1*, *PPM1N*, *PRAM1*, *S100A12*, *SIRPA*, *TMEM14A*, *UPK3A* and *ZBTB46*). From 3000 to 10,000 sorted cells were pelleted and lysed in 5 μl RLT buffer (QIAGEN) plus 1% β-mercaptoethanol, yielding 50 to 150 ng total RNA for analysis. Hybridization was performed according to the manufacturer’s instructions using a NanoString Prep Station and Digital Analyzer. nSolver Analysis software,version 3.0, was used for background correction and normalization.

### Lymphocyte proliferation assays.

Healthy volunteer T cells were prepared from healthy blood donors by immunodensity negative selection (Human T cell Enrichment Cocktail, Stem Cell Technologies, catalog 15021) and labeled with 1 μM CSFE (Invitrogen). T cells were cocultured with sorted macrophages or DCs at a 25:1 ratio for 7 days, incubated with antibodies to CD3, CD4, CD8, HLA-DR, and CD69 (Supplemental Table 3), and analyzed by flow cytometry.

### Cytokine and chemokine quantification.

Quantification was performed on medium from isolated macrophage populations stimulated ex vivo, and medium was conditioned by explanted GVHD skin and by donor-recipient MLR. For macrophage stimulation, FACS-sorted skin macrophages were stimulated with 100 ng/mL LPS from *E*. *coli* (MilliporeSigma) and harvested at 10 hours. Supernatants were cryopreserved at –80°C and batch analyzed by Luminex assay (ProcartaPlex 34-plex, eBioscience) using QIAGEN LiquiChip 200 running Luminex 100 integrated system software, version 2.3. Procartaplex Analyst, version 1.0, was used to define standard curves.

### Skin-explant assay.

Blood from BMT donor and recipient pairs was prospectively collected into EDTA before transplantation. Recipient PBMCs were irradiated (20 Gy) and used as stimulators for donor PBMCs at a 1:1 ratio. Cultures containing 1 to 5 × 10^7^ donor PBMCs were maintained in RPMI with 10% human AB serum (MilliporeSigma) for 7 days. Macrophages and T cells were sorted from MLR outputs as HLA-DR^+^CD14^+^CD11c^+^ cells and SSC^lo^CD3^+^ cells, respectively. Shave biopsies of recipient skin were taken before BMT conditioning. Biopsies were divided and incubated with medium alone (negative control), 2 × 10^5^ MLR lymphocytes, or 2 × 10^5^ MLR macrophages for 3 days in RPMI 1640 with 20% heat-inactivated autologous serum. Explants were fixed in 10% buffered formalin, sectioned, and stained with H&E. Severity of histological damage was graded using the Lerner scale by an experienced assessor blinded to experimental conditions.

### Statistics.

FlowJo, version 9.6.7, was used for analysis of flow cytometry data. PCA, hierarchical clustering, and unpaired 2-tailed *t* tests were performed in MultiExperiment Viewer software, version 4.8 (61). Reactome pathway analysis was performed in R (62). One-way ANOVA and other statistical analyses were performed using GraphPad Prism, version 7.0. *P* < 0.05 was considered significant. Additional details of statistical methods are provided in the figure legends.

### Study approval.

All human samples were obtained with informed consent according to the protocols approved by the following: Improving Haematopoietic Stem Cell Transplantation Outcome, Newcastle and North Tyneside Research Ethics Committee 2 (reference 14/NE/1136); or Newcastle Biobank, Newcastle and North Tyneside Research Ethics Committee 1 (reference 17/NE/0361).

## Author contributions

LJ, MC, and AJS conceived the project. LJ, MC, VB, and SP devised methodology. LJ, UC, MG, KG, GR, SP, MP, CAL, AKL, and VB performed experiments. LJ and XNW performed formal analysis. MC and LJ wrote the original draft of the manuscript. MC and LJ revised and edited the manuscript. LJ, MC, and AJS acquired funding. MC, EH, AP, SN, GHJ, VB, GR, and MH provided resources. MC and AJS supervised the project.

## Supplementary Material

Supplemental data

Supplemental Table 3

## Version 1 05/26/2020

In-Press Preview

## Version 2 07/27/2020

Electronic publication

## Version 3 09/01/2020

Print issue publication
