# Supplementary material for: Donor monocyte–derived macrophages promote human acute graft-versus-host disease
Source: J Clin Invest. 2020 Jul 27;130(9):4574–86. doi: 10.1172/JCI133909 (PMC7456218; doi:10.1172/JCI133909)
Supplement: Supplemental data [file jci-130-133909-s031.pdf]

Supplemental material

Table S1. BMT participant characteristics
Clinical characteristics of BMT patients with GVHD ('GVHD') and controls without GVHD ('BMT control') in skin biopsy cohort and BMT recipients undergoing BAL for IPS. Donor type: MRD= matched related donor; MUD= matched unrelated donor. Conditioning: RIC= reduced-intensity conditioning; MAC= myeloablative conditioning; TCD= T cell depleted, TR= T cell replete. Sequential conditioning was performed with FLAMSA-type protocols as described by Schmid et al. (PMID: 18176613).

	**GVHD** n=67	**BMT control** n=15
**Age**; mean (SD)	53 (12)	50 (13)
**Disease**; n (%)		
Acute leukemia	33 (49)	8 (53)
Myelodysplasia	10 (15)	1 (7)
Lymphoproliferative neoplasms	21 (32)	4 (27)
Myeloproliferative neoplasms	3 (5)	2 (13)
**Donor type**; n (%)		
MRD	19 (28)	3 (20)
MUD	45 (67)	12 (80)
Other	3 (4)	0
**HLA-match**; n (%)		
10/10	63 (94)	15 (100)
<10/10	1 (1)	0
Haploidentical	3 (4)	0
**Conditioning**; n (%)		
RIC-TCD	52 (78)	10 (67)
RIC-TR	8 (12)	1 (7)
MAC	1 (1)	1 (7)
Sequential conditioning	6 (9)	3 (20)
**Cyclosporin at biopsy**		
Serum level; μg/L mean (SD)	132 (151)	157 (127)
Proportion of biopsies acquired during cyclosporin therapy (%)	49/77 (60)	14/16 (88)

Table S2. Conditioning regimens

Group	Regimen
RIC-TCD Reduced intensity conditioning, T-depleted	Flu/Mel (Fludarabine 150mg/m^2, Melphalan 140mg/m^2, Alemtuzumab 30mg or 60mg, Cyclosporin)
	Flu/Bu (Fludarabine 150mg/m^2, Busulfan 8-10mg/kg, Alemtuzumab 30mg or 60mg, Cyclosporin)
	Flu/Bu/ATG/MMF (Fludarabine 150mg/m^2, Busulfan 9.6mg/kg, Fresenius Anti-Thymocyte Globulin 30mg/kg, Mycophenolate Mofetil, Cyclosporin)
RIC-TR Reduced intensity conditioning, T-replete	Flu/Mel/MTX (Fludarabine 150mg/m^2, Melphalan 140mg/m^2, Methotrexate 15mg/m^2, Cyclosporin)
	Flu/TBI (Fludarabine 90mg/m^2, 200cGy TBI, Methotrexate, MMF, Cyclosporin)
	RIC HAPLO (Fludarabine 150mg/m^2, Cyclophosphamide 29mg/kg, 200cGy TBI, post-transplant Cyclophosphamide 100mg/kg, Mycophenolate Mofetil, Tacrolimus)
MAC Myeloablative conditioning	Cy/TBI (12Gy TBI, Cyclophosphamide 120mg/kg, Alemtuzumab 30-60mg, Cyclosporin)
	MAC HAPLO (12Gy TBI, donor lymphocyte infusion, Cyclophosphamide 120mg/kg, Mycophenolate Mofetil, Ciclopsorin)
Sequential conditioning	FLAMSA-Bu/ATG/MMF (Fludarabine 120mg/m^2, Cytarabine 8g/m^2, Amsacrine 400mg/m^2, Fludarabine 60mg/m^2, Busulfan 9.6mg/kg, Genzyme Anti-Thymocyte Globulin 5mg/kg, Mycophenolate Mofetil, Cyclosporin)
	FLAMSA-TBI/ATG/MMF (Fludarabine 120mg/m^2, Cytarabine 8g/m^2, Amsacrine 400mg m^2, 4Gy TBI, Cyclophosphamide 80mg/kg Genzyme Anti-Thymocyte Globulin 5mg/kg, Mycophenolate Mofetil, Cyclosporin)

Table S3. Differentially expressed transcripts in monocyte to MLR-macrophage transition.
See supplementary data file showing the mean fold changes, p value (unpaired t test) and FDR for three pairs of monocyte and MLR-macrophage data. Bold type indicates genes also upregulated in GVHD macrophages in vivo

Table S4. Antibodies

REAGENT or RESOURCE	SOURCE	IDENTIFIER
**Antibodies**		
CD3 unconjugated	BD	555330; UCHT1
CD11c biotin	BD	555391; B-ly6
Factor XIIIa unconjugated	Enzyme Research	SAF13A-AP
Donkey anti-mouse Alexa Fluor 488	Jackson	715-545-150
Donkey anti-sheep Alexa Fluor 647	Invitrogen	A-21448
Streptavidin Cy5	Jackson	016-170-084
CD45 APCCy7	BD	557833
CD3 PERCPCy5.5	BD	332771
CD4 BV421	BioLegend	300531
CD8 PE	BD	345773
CD127 APC	BioLegend	351315
CD25 FITC	BD	345796
CD45 V500	BD	560777
CD45 Alexa Fluor 700	BioLegend	304024
HLA-DR V500	BD	561224
HLA-DR Alexa Fluor 700	BD	560743
HLA-DR V450	BD	642276
CD14 BV650	BD	301835
CD1c PECy7	BioLegend	331506
CD11c APCCy7	BioLegend	337218
CD11c V450	BD	560369
CD123 PERCPCy5.5	BD	558714
CD141 PE	BD	559781
CD141 APC	Miltenyi	130-090-907
CD3 FITC	BD	345763
CD4 PE	BD	555347
CD16 PE-Dazzle594	BioLegend	302054
CD14 APCCy7	BD	557831
CD14 FITC	BD	555497
CD1c APC	BD	559775
CD11c APC	BD	559877
HLA-DR FITC	BD	556643
CD86 PE	BD	555658
CD209 FITC	BD	551264
CD163 APC	R&D	FAB16078
CD16 FITC	BD	335035
CD206 APC	BD	550889
CD64 PE	BioLegend	305007
CD172a PE	BioLegend	323805
CD3 V500	BD	561416
CD4 PE	BD	55347
CD8 APCCy7	BD	557834

HLA-DR PERCPCy5.5	BD	339126
CD69 PE-Cy7	BD	557745
CD3 FITC	BD	345763
CD19 FITC	BD	345776
CD20 FITC	BD	345792
CD56 FITC	BD	345811
HLA-DR V500	BD	561224
CD14 Qdot655	Invitrogen	Q10056
CD16 APCH7	BD	560195
CD45 FITC	BD	345808
Annexin V PE	BD	56-65875X
7-AAD	BD	56-66121E
S100A8/9 PE	Novus	NBP2-2526PE

Figure S1

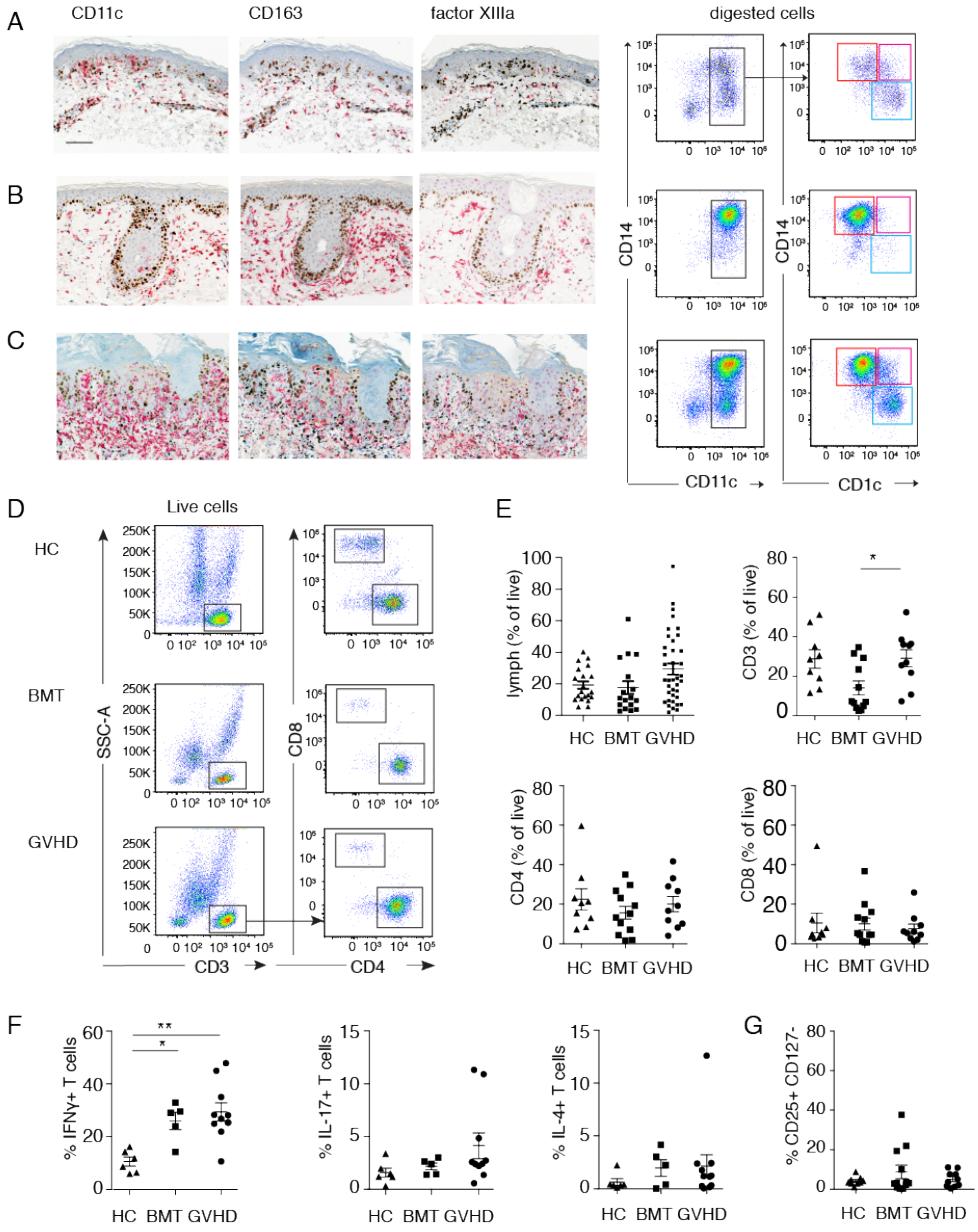
A
CD11c
CD163
factor XIIIa
digested cells
B
C
CD14
CD11c
CD1c
D
Live cells
HC
BMT
GVHD
SSC-A
CD3
CD8
CD4
E
lymph (% of live)
CD3 (% of live)
CD4 (% of live)
CD8 (% of live)
HC BMT GVHD
F
% IFNγ+ T cells
% IL-17+ T cells
% IL-4+ T cells
G
% CD25+ CD127-

Fig. S1. Additional characterization of the GVHD infiltrate.

A-C. Paired IHC and flow cytometry analysis of acute GVHD showing increasing burden of macrophages with consistent flow phenotypes. GVHD biopsies were immunostained with antibodies to CD11c and CD163 and factor XIIIa as indicated (red) and co-stained with antibodies to Ki67 (brown). Note epidermal stress indicated by the proliferation marker Ki-67. Biopsies were all reported as histological grade II GVHD occurring at: a) 56 days; b) 214 days; c) 278 days. Paired flow cytometry analysis was performed as described in Figure 1. Although these selected biopsies suggest an increase in macrophage burden with time post-transplant, no significant relationship was observed when all quantitative data were analysed together (not shown).
D. Gating strategy for lymphocytes in flow cytometry of single cell suspensions of digested dermis from patients with acute GVHD, BMT controls and healthy controls. Among live cells, CD3+ SSC^{lo} lymphocytes were divided by CD4 and CD8 expression. Representative examples from more than 8 experiments are shown.
E. Relative abundance of lymphocytes by flow cytometry of single cell suspensions of digested dermis from healthy controls (HC; n = 9), BMT controls without GVHD (BMT; n = 8), or patients with acute GVHD (GVHD; n = 10). Error bars show mean ± SEM. * $p<0.05$ by one-way ANOVA with Dunnett's multiple comparisons test. Lymph = cells falling in the HLA-DR- SSC low lymphoid gate (see Figure 1).
F. Cytokine production by CD3+ T lymphocytes from healthy control (HC, n=6), BMT transplant control (BMT, n=5) or patients with GVHD (GVHD, n=10), following ex vivo stimulation by PMA and ionomycin assessed by flow cytometry. Error bars show mean ± SEM. * $p<0.05$ and ** $p<0.01$ by one-way ANOVA with Dunnett's multiple comparisons test.
G. Proportion of CD25+ CD127- cells among CD4+ T lymphocytes from healthy control (HC, n=6), BMT transplant control (BMT, n=5) or patients with GVHD (GVHD, n=10).

Figure S2

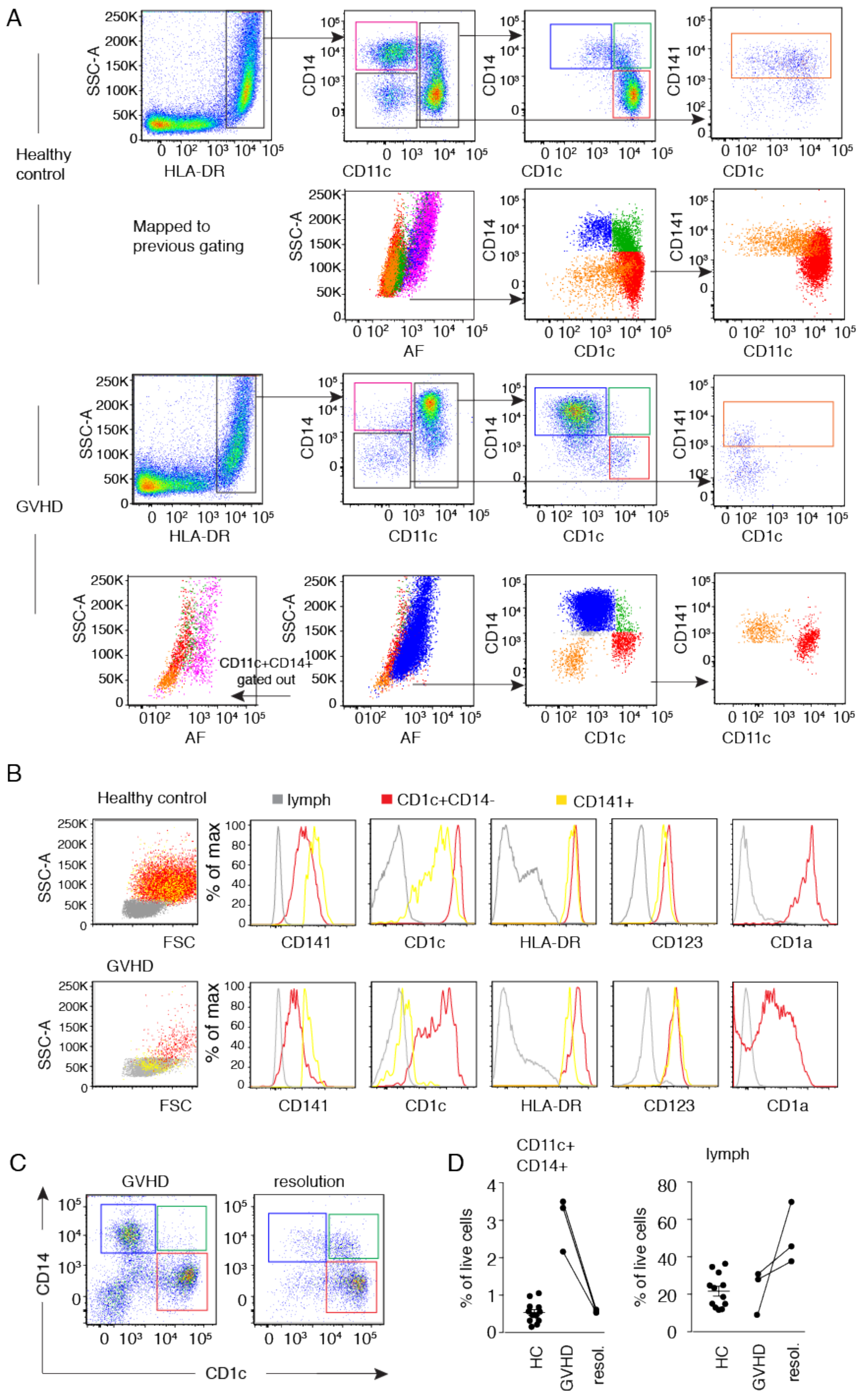
A
Healthy control
Mapped to previous gating
GVHD
CD11c+CD14+ gated out
SSC-A
HLA-DR
CD14
CD11c
CD1c
CD141
AF
B
Healthy control
lymph
CD1c+CD14-
CD141+
GVHD
% of max
FSC
CD141
CD1c
HLA-DR
CD123
CD1a
C
GVHD
resolution
CD14
CD1c
D
CD11c+ CD14+
lymph
% of live cells
HC
GVHD
resol.

Fig. S2. Gating strategy for characterization of mononuclear cells in GVHD
A Comparison of previous gating method using autofluorescence to identify dermal macrophages with current schema used to identify myeloid cells in the healthy control and GVHD dermis.
Healthy control dermis: Top row shows populations identified with the gating scheme used in Figure 1. Bottom row shows projections of these populations onto the gating scheme used previously, in which autofluorescence was used to identify macrophages (pink) and the small population of monocyte-derived macrophages in normal skin forms a small population (blue) at the interface between macrophages and DC (CD1c+ cDC2: red; CD141+ cDC1: orange). CD14+CD1c double positive cells: green.
GVHD skin: Top row shows populations identified with the gating scheme used in Figure 1. Bottom row shows projections of these populations onto the gating scheme used previously. Note that the dominant population of monocyte-dervide GVHD macrophages (blue) obscures the distinction between macrophages and DC on the autofluorescence axis. Resident macrophages (pink) can be recovered after the CD11c+ CD14+ GVHD macrophages are gated out (bottom row left-most panel).
B. Scatter properties and selected antigen expression, confirming the identity of CD1c+ cDC2 (red) and CD141+ cDC1 (yellow) populations comparing healthy control dermis and GVHD dermis.
C. Mononuclear cell analysis in paired biopsies at GVHD onset and following resolution of skin inflammation. Dot plots show CD11c+ cells in a representative patient at GVHD presentation and follow-up 86 days later.
D. Proportion of CD11c+ CD14+ GVHD macrophages and lymphocytes at presentation of GVHD and follow-up in 3 paired samples quantified as % of live cells. Accompanying normal ranges in healthy control skin measured by flow cytometry are shown as dot plots with mean ± SEM (n=15).

Figure S3

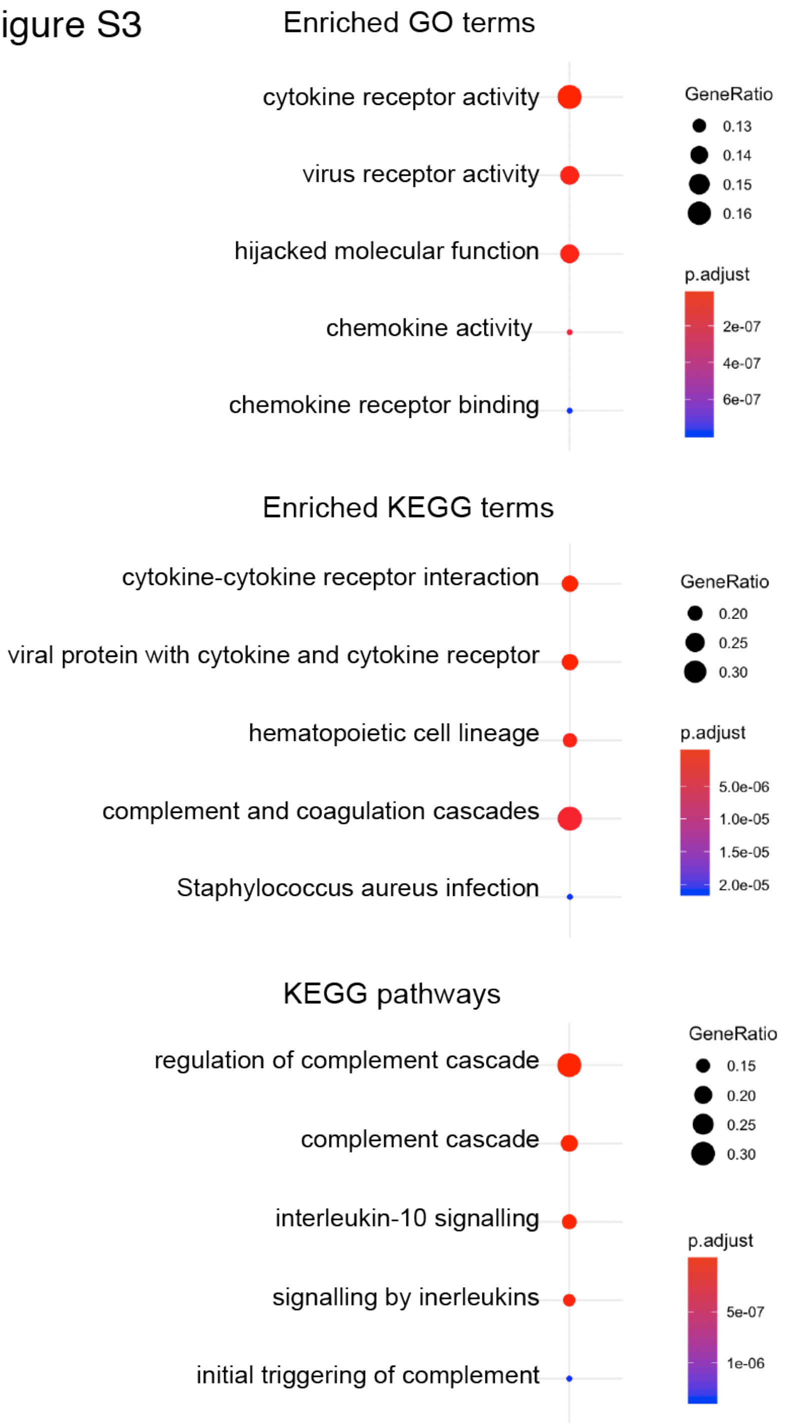

Fig. S3. Functional annotation of monocyte to macrophage transition in mixed leukocyte reactions

The top 50 most differentially expressed genes comparing monocytes (n = 3) and their transition to macrophages differentiated in an HLA-matched mixed leukocyte reaction (n = 3). Functional annotations were performed in DAVID. For a full list of genes please refer to Table S4.
